# Neutropenic Enterocolitis Following Autologous Stem Cell Transplantation: A Compelling Clinical Case Report Written With the Assistance of ChatGPT

**DOI:** 10.7759/cureus.36390

**Published:** 2023-03-20

**Authors:** Vanessa Milan-Ortiz, Anirudh R Damughatla, Adam M Qazi, Saivaishnavi Kamatham, Sharad Oli, Pragna Koleti, Diane L Levine

**Affiliations:** 1 Internal Medicine, Wayne State University Detroit Medical Center, Detroit, USA; 2 Pulmonary Critical Care, Wayne State University Detroit Medical Center, Detroit, USA; 3 Internal Medicine, Suburban Community Hospital, Norristown, USA

**Keywords:** post-autologous stem cell transplantation enterocolitis, enterocolitis, artificial intelligence, neutropenic enterocolitis, chatgpt

## Abstract

Neutropenic enterocolitis (NE) is a rare and life-threatening condition that typically occurs in patients with hematologic malignancies undergoing intensive chemotherapy, radiation therapy, or bone marrow transplant regimens, predisposing them to profound neutropenia. NE can have a nonspecific clinical presentation and mimic other gastrointestinal disorders such as appendicitis, diverticulitis, or inflammatory bowel disease but is associated with very high morbidity and mortality if not diagnosed and treated promptly. We present the case of a middle-aged female with a recent diagnosis of follicular lymphoma who developed neutropenic enterocolitis after undergoing induction chemotherapy for an autologous stem cell transplant (ASCT). In this article, we provide a literature review of neutropenic enterocolitis and highlight the importance of a prompt diagnosis and management, given its high mortality rate.

## Introduction

Neutropenic enterocolitis (NE), also called typhlitis, poses a severe threat to individuals with compromised immune systems. This life-threatening condition is distinguished by the extensive, circumferential thickening of the proximal colon, primarily impacting the cecum. The precise pathophysiological mechanisms driving NE are still not comprehensively understood, but it is thought to stem from damage to the intestinal mucosa caused by cytotoxic chemotherapy, radiation, or leukemic infiltration [1]. Left untreated, the mortality rate of NE ranges from 50% to 100% [2,3], underscoring the urgent need for a prompt, accurate diagnosis and management. To further elucidate the clinical features of NE in a patient population, we present a compelling case of a middle-aged female who developed NE following induction chemotherapy for an autologous stem cell transplant (ASCT).

## Case presentation

A 53-year-old female with a history of follicular lymphoma was admitted for an ASCT. This patient was initially diagnosed with follicular lymphoma a year before admission when she presented with a right groin mass. Imaging revealed multiple lymph nodes in the right common iliac, external iliac, and inguinal region distribution. An excisional biopsy confirmed low-grade lymphoma with positive CD10 of germinal B-cell origin, and the patient achieved complete disease remission following three cycles of bendamustine and rituximab. However, several years later, a surveillance positron emission tomography-computed tomography (PET-CT) scan indicated disease progression in the right calf area, and a tibia bone biopsy confirmed lymphoma recurrence. The patient received three cycles of rituximab plus cyclophosphamide, doxorubicin, vincristine, and prednisone (R-CHOP), resulting in complete remission.

**Figure 1:**
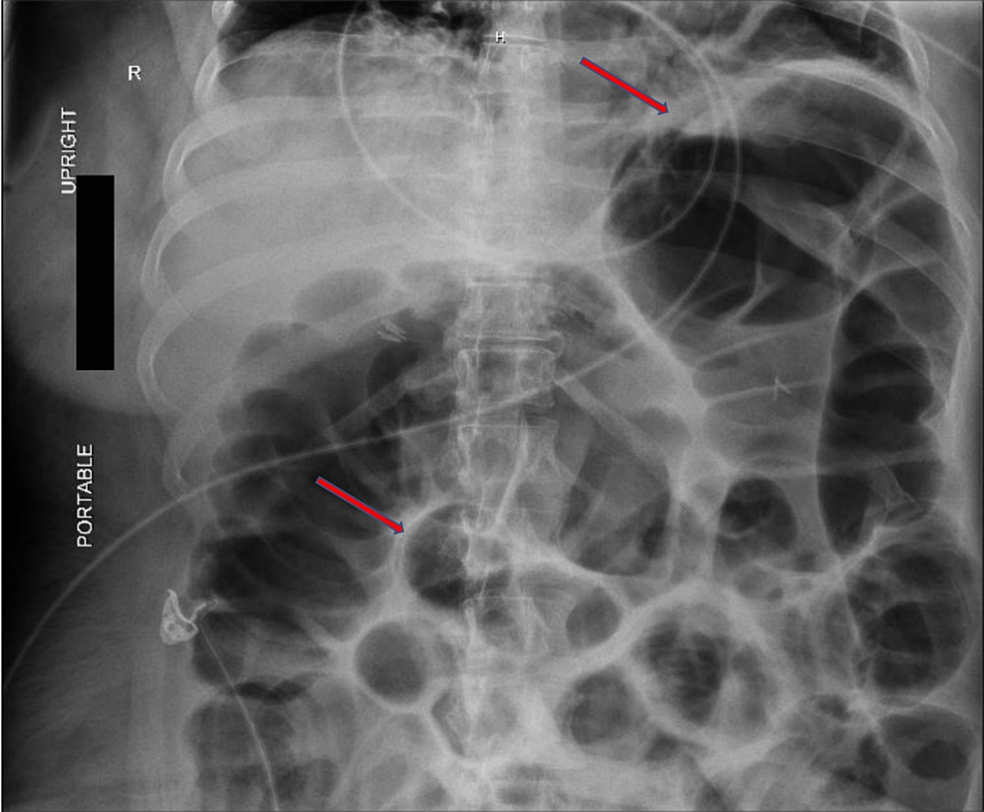
Abdominal X-ray demonstrating small and large bowel distention (red arrows).

**Figure 2:**
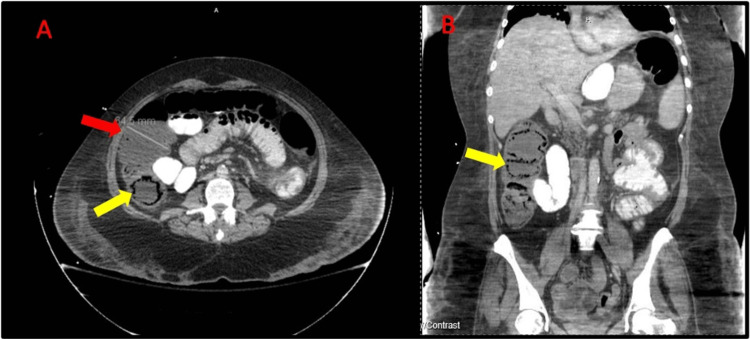
A: Initial CT (axial view) of the abdomen demonstrating pneumatosis coli (yellow arrow) and colonic distension (red arrow). B: CT scan (coronal view) showing pneumatosis coli of the ascending colon (yellow arrow). CT: computed tomography

**Figure 3:**
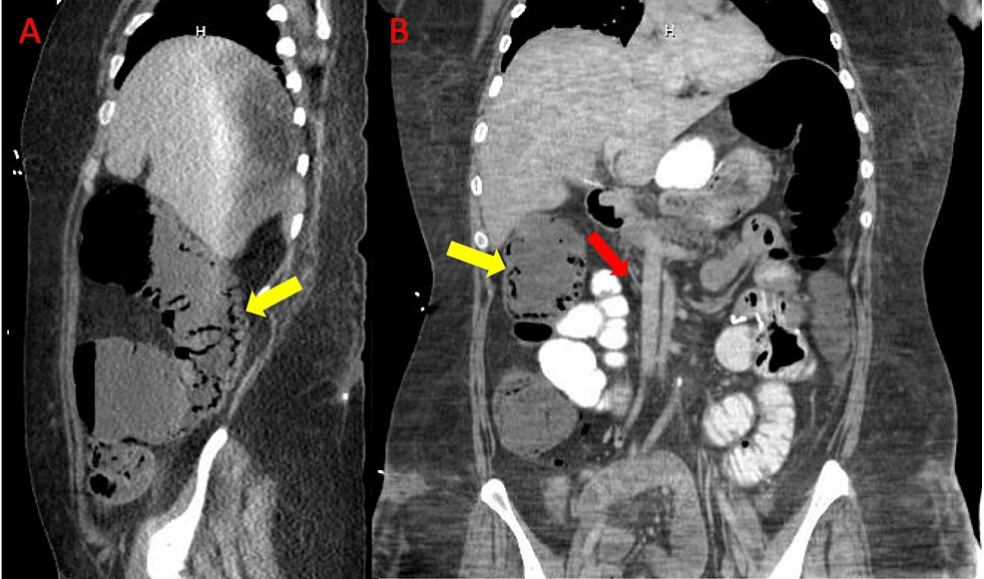
A: CT (sagittal view) redemonstrating pneumatosis coli of the ascending colon (yellow arrow). B: CT (coronal view) demonstrating gas in the mesenteric veins (red arrow). CT: computed tomography

During her ASCT preparative regimen with carmustine, etoposide, cytarabine, and melphalan (BEAM), the patient experienced severe complications. On the fifth day of hospitalization, she became hypotensive and febrile and developed 6-8 watery bowel movements. Her condition rapidly deteriorated, and she was transferred to the intensive care unit, where she was treated with vasopressors and broad-spectrum antibiotics for febrile neutropenia. Stool studies were negative for infectious agents. On abdominal X-ray, the patient was found to have distended small and large bowels (Figure 1), and an abdominal/pelvic computed tomography (CT) scan revealed pneumatosis coli with mesenteric venous and intrahepatic portal veins gas, indicative of colonic necrosis and severe nonspecific adynamic ileus (Figure 2A-2B and Figure 3A-3B). Laboratory results revealed pancytopenia with a WBC count of <0.1 × 10^9^/L (absolute neutrophil count: 0.3 × 10^9^/L), indicating severe neutropenia.

**Figure 4:**
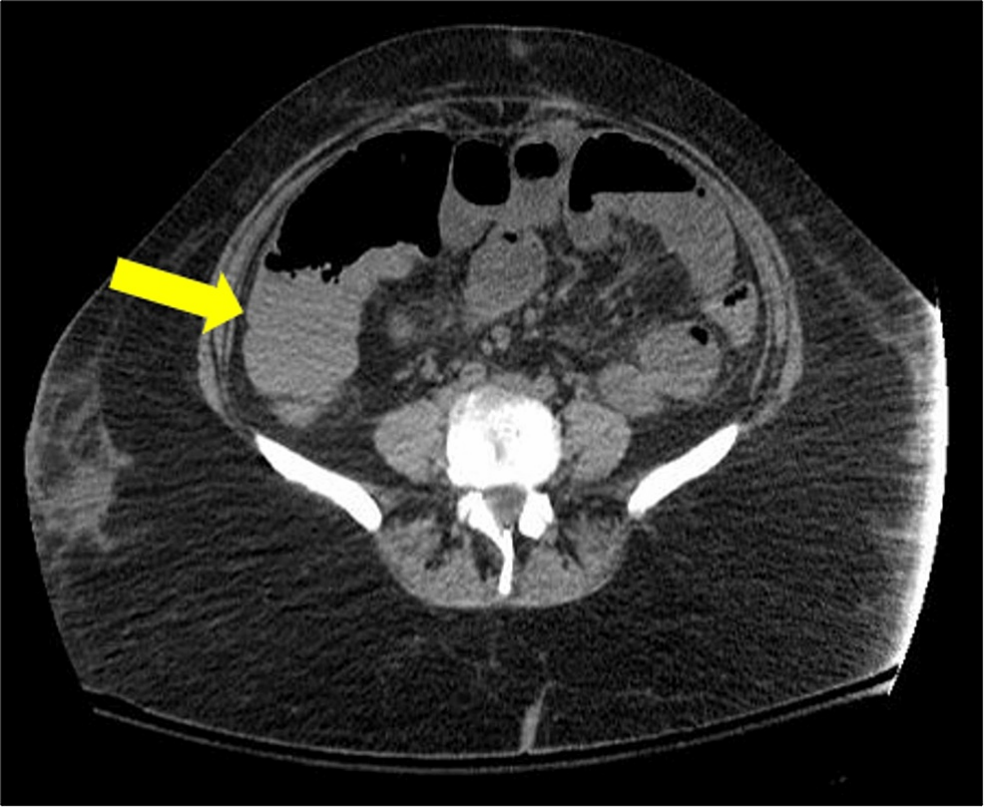
Posttreatment follow-up abdominal CT scan (axial view) that demonstrates resolution of pneumatosis coli with persistent distended bowel loops (yellow arrow). CT: computed tomography

The patient was treated with broad-spectrum antibiotics, antifungals, and antivirals, and repeat imaging showed a near-complete resolution of pneumatosis coli of the right colon (Figure 4). Our patient recovered quickly due to prompt diagnosis and treatment; therefore, no surgical intervention had to be carried out.

## Discussion

NE is a rare but potentially life-threatening condition that occurs most commonly in neutropenic patients. The prevalence of NE varies among different patient populations, with reports ranging from 0.8% to 26% [4]. Gorschlüter et al. reported a 5.3% pooled incidence rate of NE in adults hospitalized for the treatment of hematologic malignancies or solid tumors, although noting this figure to be an underestimate [4]. NE is more common in patients with hematologic malignancies, particularly those with acute myeloid leukemia and non-Hodgkin lymphoma who receive intensive chemotherapy regimens [5]. However, NE has been reported in association with several chemotherapy regimens, including those that contain cytarabine, anthracyclines, and platinum agents [2]. NE may also occur in patients receiving radiation therapy or immunosuppressive therapy. The incidence of NE is also higher in patients with prolonged neutropenia, as well as those with additional risk factors such as hypotension, sepsis, and the use of antibiotics.

NE has also been reported in patients with other underlying conditions that cause neutropenia and immune suppression, such as severe infections, autoimmune diseases, and bone marrow failure syndromes [5]. Additionally, NE has been observed in patients receiving immunosuppressive therapy for organ transplantation and those with congenital neutropenia or cyclic neutropenia. The exact mechanism by which NE develops in these conditions is not well understood, but it is thought to result from injury to the intestinal mucosa due to a combination of factors such as cytotoxic agents, immunosuppression, and altered gut flora [5]. The clinical presentation and management of NE may differ depending on the underlying condition and the severity of the neutropenia.

The diagnosis of NE can be challenging, as symptoms can be nonspecific and overlap with other common complications of chemotherapy, such as infectious diarrhea and neutropenic fever. However, early recognition of NE is critical, as prompt diagnosis and treatment can significantly improve outcomes [1]. Conservative management, which includes bowel rest, parenteral nutrition, and broad-spectrum antibiotics, is the standard of care for most patients with NE and has been shown to improve outcomes and reduce the need for surgery. In addition to bowel rest and broad-spectrum antibiotics, supportive care may include aggressive fluid resuscitation, blood product transfusions, and close monitoring for complications such as perforation or sepsis.

Surgical intervention in NE is not always required and should be considered a last resort in patients who do not respond to conservative management or develop complications such as perforation, abscess, or uncontrolled bleeding. The decision to perform surgery is based on the clinical presentation, severity of symptoms, and imaging findings. In a study of 329 patients with NE, conservative management was successful in 69% of cases, and only 31% required surgery [2]. Another study reported a success rate of 80% for conservative management, with surgery needed in 20% of cases [6].

Untreated NE has a high mortality rate ranging from 50% to 100%, which is why early recognition and management are essential for improving patient outcomes [7,8]. With appropriate treatment, the mortality rate decreases to 30%-50% [7,8]. Mortality is often attributed to complications such as sepsis, bowel perforation, and multi-organ failure. The mortality risk is higher in patients with more severe disease, longer duration of neutropenia, older age, and comorbidities. The use of prophylactic antibiotics in neutropenic patients with cancer has also been shown to reduce the incidence of NE and the need for surgical intervention. However, the role of prophylactic antibiotics in patients undergoing autologous stem cell transplant is still controversial and requires further study.

Given the high mortality rate of NE, physicians caring for neutropenic patients should have a low threshold for considering NE in the differential diagnosis of gastrointestinal symptoms, particularly in patients receiving intensive chemotherapy or ASCT. In our patient's case, NE was diagnosed based on imaging findings and clinical presentation. The successful management of NE in our patients highlights the importance of early diagnosis and prompt, targeted interventions in improving clinical outcomes in this challenging population.

**Figure 5:**
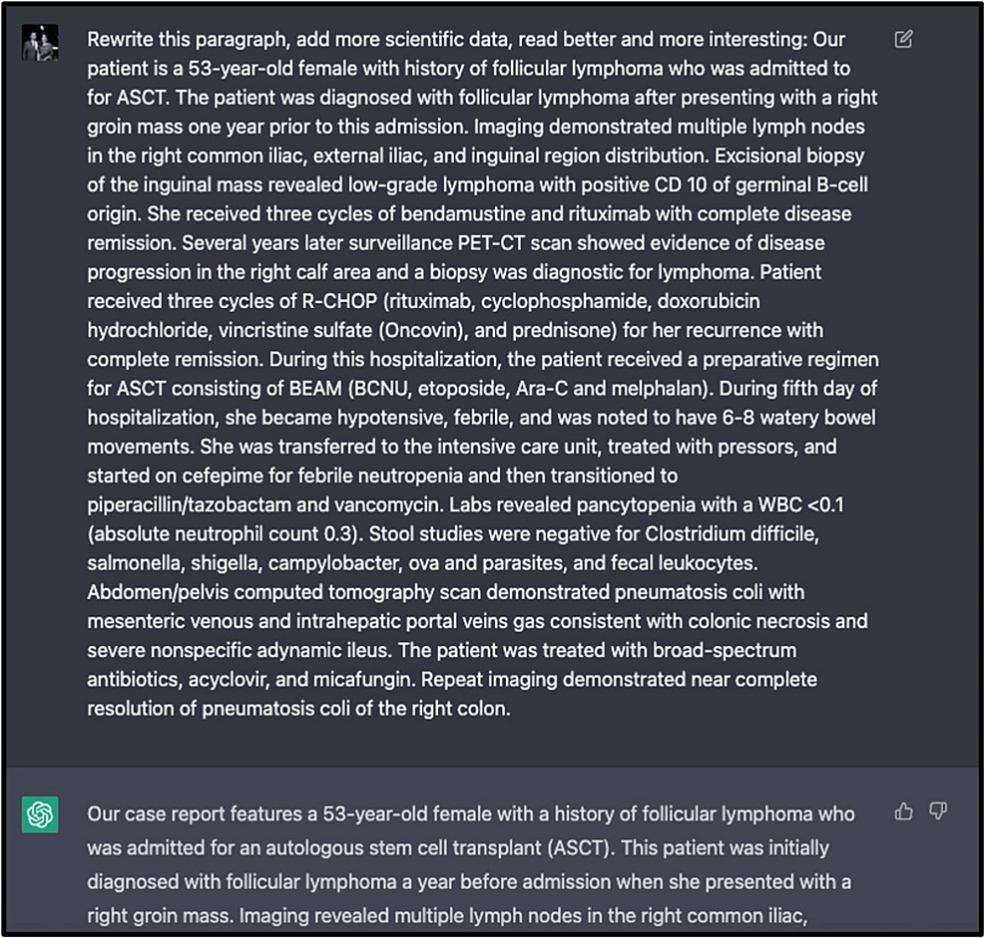
Screenshot #1 showing how ChatGPT is used in writing the case report.

**Figure 6:**
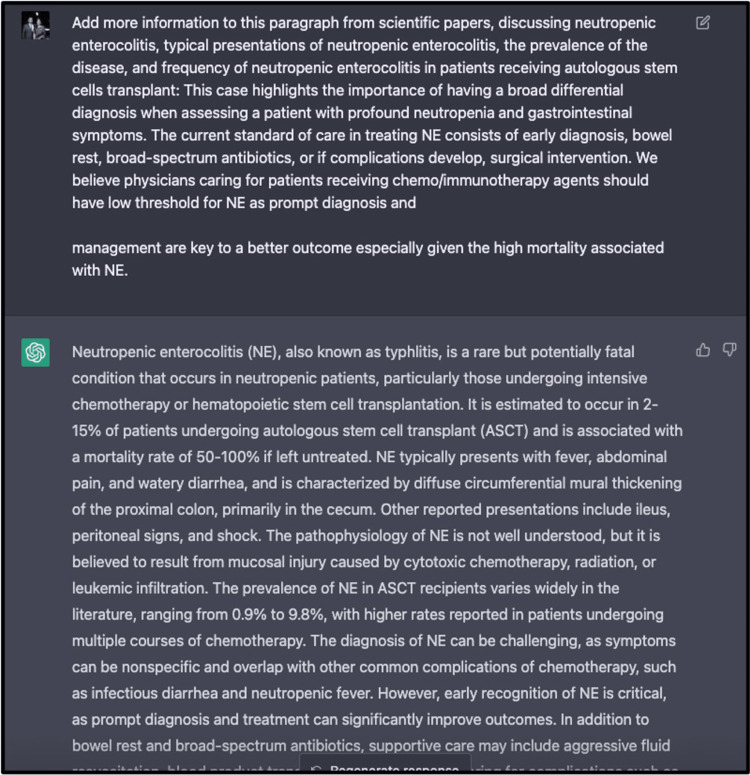
Screenshot #2 showing how ChatGPT is used in writing the case report.

Below are screenshots (Figure 5 and Figure 6) showing the utilization of ChatGPT to produce comprehensive insights into the disease and to enhance the language and structure of the existing case report's paragraphs.

## Conclusions

In conclusion, this case report highlights the challenges that can arise during ASCT for patients with a history of follicular lymphoma. The patient in this report developed hypotension, febrile neutropenia, and diarrhea, which was later confirmed with the diagnostic imaging to be NE and resolved following prompt and effective treatment. This case underscores the importance of close monitoring and prompt intervention for patients undergoing ASCT and the potential for serious complications related to febrile neutropenia and abdominal issues. This information is valuable for healthcare providers as they care for patients undergoing ASCT and strive to minimize the risk of complications. Further research is needed to determine the best strategies for preventing and managing these complications in patients undergoing ASCT.
